# Simultaneous imaging of GFP, CFP and collagen in tumors *in vivo *using multiphoton microscopy

**DOI:** 10.1186/1472-6750-5-14

**Published:** 2005-05-23

**Authors:** Erik Sahai, Jeffrey Wyckoff, Ulrike Philippar, Jeffrey E Segall, Frank Gertler, John Condeelis

**Affiliations:** 1 Tumour Cell Biology Laboratory, Cancer Research UK London Research Institute, London WC2A 3PX, UK; 2 Department of Anatomy and Structural Biology, Albert Einstein College of Medicine, 1300 Morris Park Avenue, Bronx, NY 10461, USA; 3 Department of Biology, Massachusetts Institute of Technology, 77 Massachusetts Avenue, Cambridge, MA 02139, USA; 4 Analytical Imaging Facility, Albert Einstein College of Medicine, 1300 Morris Park Avenue, Bronx, NY 10461, USA

## Abstract

**Background:**

The development of multiphoton laser scanning microscopy has greatly facilitated the imaging of living tissues. However, the use of genetically encoded fluorescent proteins to distinguish different cell types in living animals has not been described at single cell resolution using multiphoton microscopy.

**Results:**

Here we describe a method for the simultaneous imaging, by multiphoton microscopy, of Green Fluorescent Protein, Cyan Fluorescent Protein and collagen *in vivo *in living tumors. This novel method enables: 1) the simultaneous visualization of overall cell shape and sub-cellular structures such as the plasma membrane or proteins of interest in cells inside living animals, 2) direct comparison of the behavior of single cells from different cell lines in the same microenvironment *in vivo*.

**Conclusion:**

Using this multi-fluor, multiphoton technique, we demonstrate that motility and metastatic differences between carcinoma cells of differing metastatic potential can be imaged in the same animal simultaneously at sub-cellular resolution.

## Background

The ability to image cell movements and dynamic changes in sub-cellular structures in living mammals will significantly enhance our understanding of biology. Many applications have been developed to investigate how tumor cells act *in vivo *[1-3]. In recent years multi-photon laser scanning microscopy has demonstrated that it has both the resolution and tissue penetration to significantly improve the analysis of tumor cell behavior in vivo [4-6]. A key requirement for multiphoton microscopy is the need to fluorescently label cells, sub-cellular compartments or proteins of interest. Green Fluorescent Protein (GFP) has been widely used to label cells; however the use of a single fluorophore, though successful, has been limiting. Tumor development and cell migration from intravasation to metastatic growth has been studied *in vivo *in orthotopic models and transgenic mice [7] using GFP, but limited by the fact that only one cell type can be examined without introducing a dye from an external source. GFP and RFP have been used to study separate cell populations in tumors using conventional imaging methods [8-10] and multiple flours have been used in cells *in vitro *[11]. However, this combination of fluorophores are not compatible with multiphoton intravital imaging, since the high intensity pulsed infrared lasers commonly used for multiphoton microscopy produce light in the 720–980 nm range and are unable to excite RFP efficiently in deep tissue. While we and other groups have been able to use multiphoton laser scanning microscopy to image two or more chromophores *in vivo*, generally non genetically coded fluors such as Texas Red-labeled dextran or Hoechst are used [6,12-15] in conjunction with GFP or other fluorescent proteins which prevents the application of multiphoton microscopy from being applied to the study of multiple cell populations in vivo. Therefore, here we describe methods to image the genetically encoded fluors GFP and Cyan Fluorescent Protein (CFP) simultaneously in a living tissue. Previous work by our group has correlated patterns in gene expression in cells with differing metastatic potential with differences in cell motility and polarization *in vivo *[6,16]. Here we describe a method to compare the behavior of cancer cells in which expression of genes identified in these studies has been altered with the behavior of control cells in the same tumor micro-environment. We also describe a method for imaging two genetically encoded fluorophores in the same cell, thereby allowing imaging of sub-cellular compartments or proteins and the whole cell simultaneously.

## Results and Discussion

To study two cell types in the same organ *in vivo *with differing fluorescent proteins, fluorescent pairs need to be chosen that can be excited equally at a common wavelength. We chose to simultaneously image GFP and CFP because their expression is well tolerated by most cell types and they are easily excited by standard Ti-sapphire lasers. Initial attempts to image RFP were not successful; both tetrameric and dimeric variants of dsRed formed aggregates which had deleterious effects on cell viability. The use of monomeric mRFP overcame these problems, however the power output of most Ti-Sapphire lasers drops off significantly at the wavelengths of light required to excite mRFP resulting in ineffective excitation of mRFP. While YFP has been used as part of a FRET pair *in *vivo [17], GFP was chosen over YFP because YFP is not effectively excited by wavelengths below 900 nm [18], YFP and GFP excitation closely mirror each other and are difficult to separate, and a large number of already available cell lines and transgenic animals use GFP.

**Figure 1:**
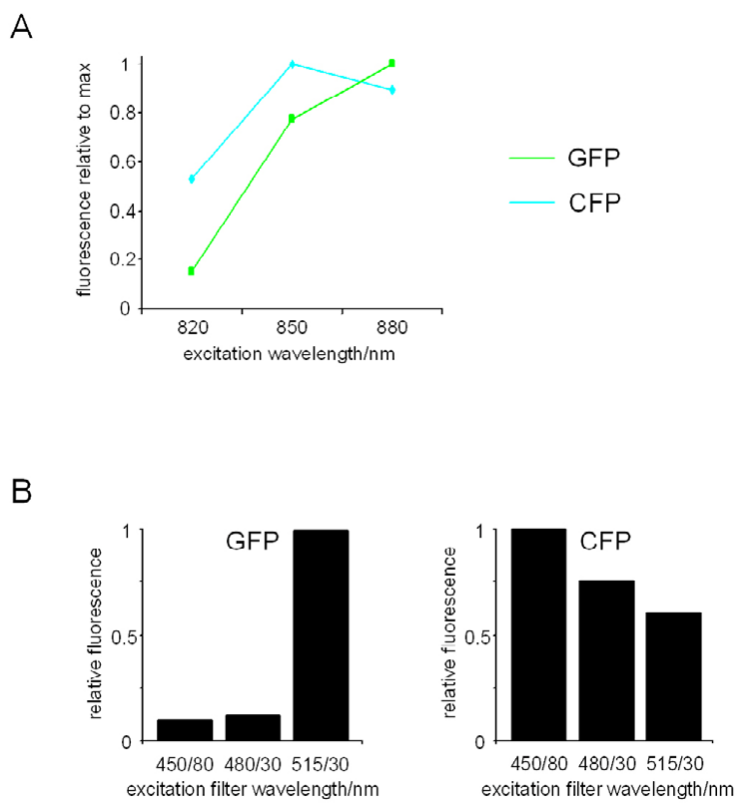
2-photon excitation and emission properties of GFP and CFP expressed in cells. A) Stable cell lines expressing either CFP or GFP were imaged with the indicated excitation wavelength (in all cases the laser power was 0.95–1.0W). CFP fluorescence was captured using a 480/30 filter and non-descanned detection, GFP fluorescence was captured using a 515/30 filter and non-descanned detection. The amount of fluorescence captured using different excitation wavelengths is shown (relative to the maximum fluorescence). B) Stable cell lines expressing either CFP or GFP were imaged using an 880 nm excitation beam (in all cases the laser power was 0.95–1.0W). Fluorescence was captured using the indicated filters and non-descanned detection. The amount of GFP or CFP fluorescence captured using different filters is shown (relative to the maximum fluorescence).

Using a Biorad Radiance 2000 multiphoton microscope with an inverted Olympus IX70 connected to a Spectra Physics Tsunami Ti-Sapphire laser, we first determined the best 2-photon excitation wavelength by measuring the fluorescence emitted by GFP and CFP when excited by different wavelengths. The excitation of CFP was most effective around 850 nm and declined at longer wavelengths, whereas excitation of GFP was still increasing at 880 nm with a maximum at 960 nm (Figure 1a and data not shown). These data are in agreement with previously described excitation cross sections for GFP and CFP ( and [18]). In conclusion, wavelengths around 880 nm were most suitable for simultaneous imaging of GFP and CFP because 880 nm is very close to the optimal wavelength for exciting CFP and is also able to excite GFP.

**Figure 2:**
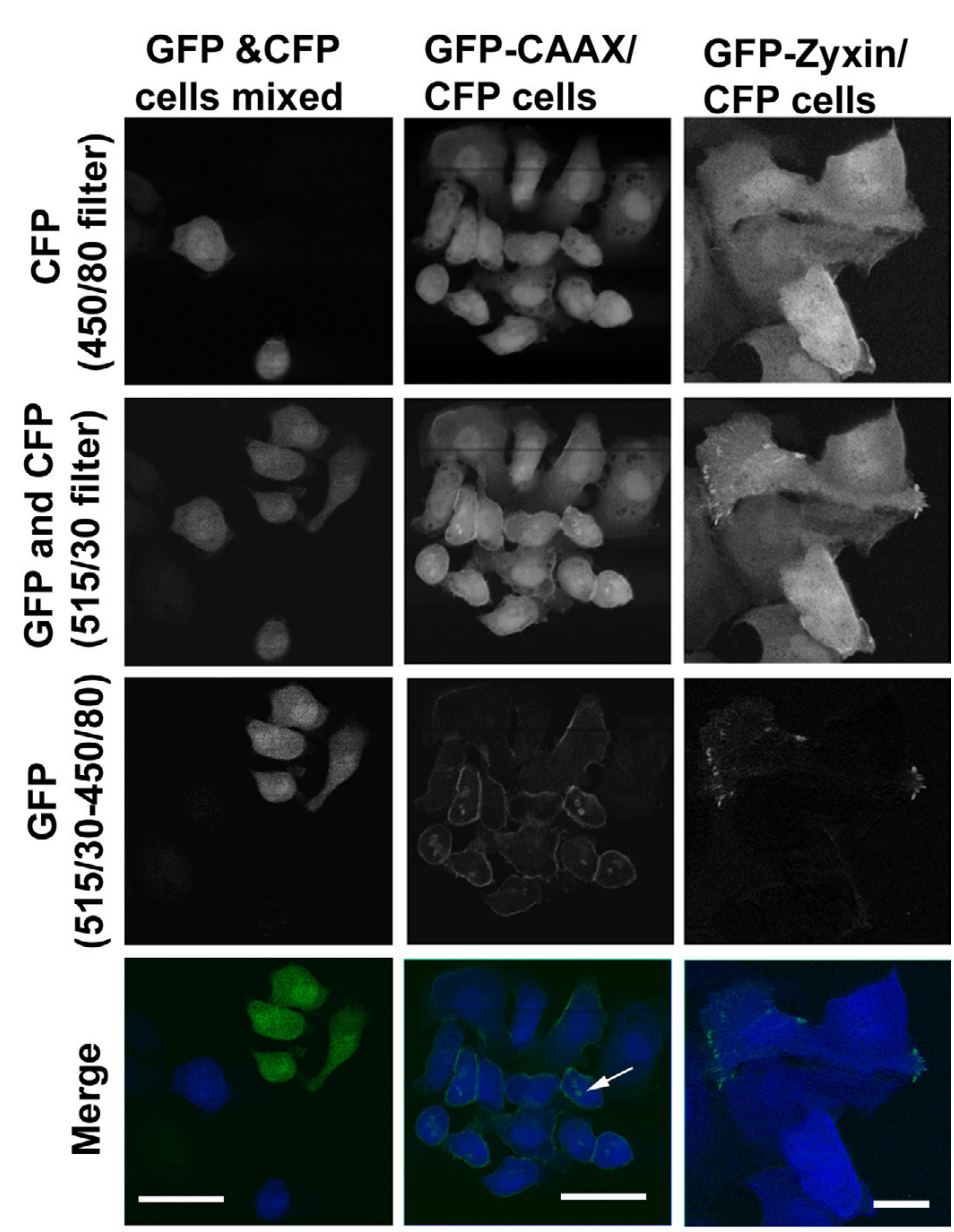
Simultaneous capture of GFP and CFP. Stable cell lines expressing either GFP or CFP were co-cultured (left panels) or a stable cell line expressing CFP and GFP-CAAX (membrane targeted – middle panels) or a cell line expressing CFP transiently transfected with GFP-ZYXIN (right panels) were analyzed. Top panels show the fluorescence captured using a 450/80 filter and non-descanned detection. Upper-mid panels show the fluorescence captured using a 515/30 filter and non-descanned detection. Lower-mid panels show the result of subtracting the 450/80 signal from the 515/30 signal. Bottom panels show a merge of the top panels (CFP signal in blue) and the lower-mid panels (GFP signal in green). GFP-CAAX, which generally targets to the membrane can be seen labeling of intracellular structures in the merge and GFP images (middle panel; arrow). All images were taken with a 40× objective and a final magnification of 500× for the right 2 columns and 1000× for the left. Scale bar = 25 μm for the right 2 columns and 10 μm for the left.

Having established that both GFP and CFP can be effectively excited at 880 nm we then sought establish a simple method for separating the fluorescence emitted by the different proteins. We tested three filters for their ability to pass light emitted by cells expressing either GFP or CFP. Figure 1B shows that GFP fluorescence passed through the 515/30 nm filter (median wavelength/band width) but did not pass through the 450/80 nm and 480/30 nm filters. In contrast, CFP fluorescence passed efficiently through all three filters. Therefore, both GFP and CFP fluorescence will pass through a 515/30 filter whereas 450/80 and 480/30 filters will only pass CFP fluorescence. Subtraction using the 'Image Calculator' feature on Image J (available from ) of the CFP fluorescence captured using either a 450/80 or 480/30 filter from the mixture of GFP and CFP fluorescence passing through a 515/30 filter should yield the GFP fluorescence. To test this we tried to image either co-cultures of GFP and CFP expressing cells or cells co-expressing GFP and CFP constructs. To facilitate the subtraction we adjusted the gain settings during image acquisition to ensure that a CFP expressing cell produced the same pixel intensity when captured using either the 450/80, 480/30 or 515/30 filters. Figure 2 shows that subtraction of the image captured using the 450/80 filter from that captured the 515/30 filter leaves only the GFP expressing cells visible (Figure 2 left hand panels). Furthermore, if the same subtraction is performed on images of cells co-expressing GFP targeted to the plasma membrane (GFP-CAAX) and CFP in the cytoplasm. After subtraction of the CFP signal, the distribution of GFP signal is indistinguishable from GFP images of cells expressing only GFP-CAAX (Figure 2 middle panels and data not shown). GFP-CAAX also localized to some intracellular structures; it is possible that this may be the Golgi apparatus as Michaelson *et al *have reported that a similar construct localized to both the plasma membrane and the Golgi apparatus [19].

To test if this method can be used to monitor protein localization, we transiently transfected cells expressing CFP with a GFP-ZYXIN expression construct. Figure 2 (right hand panels) shows one such transfected cell amongst other non-transfected cells, subtraction of the 450/80 signal from the 515/30 signal clearly reveals the focal adhesion localization of GFP-ZYXIN in the transfected cell while the untransfected cells are not seen. These results demonstrate that GFP and CFP fluorescence can be successfully separated even when simultaneously excited using an 880 nm laser beam. Furthermore, specific sub-cellular compartments or proteins can be visualized in cells simultaneously expressing GFP and CFP.

**Figure 3:**
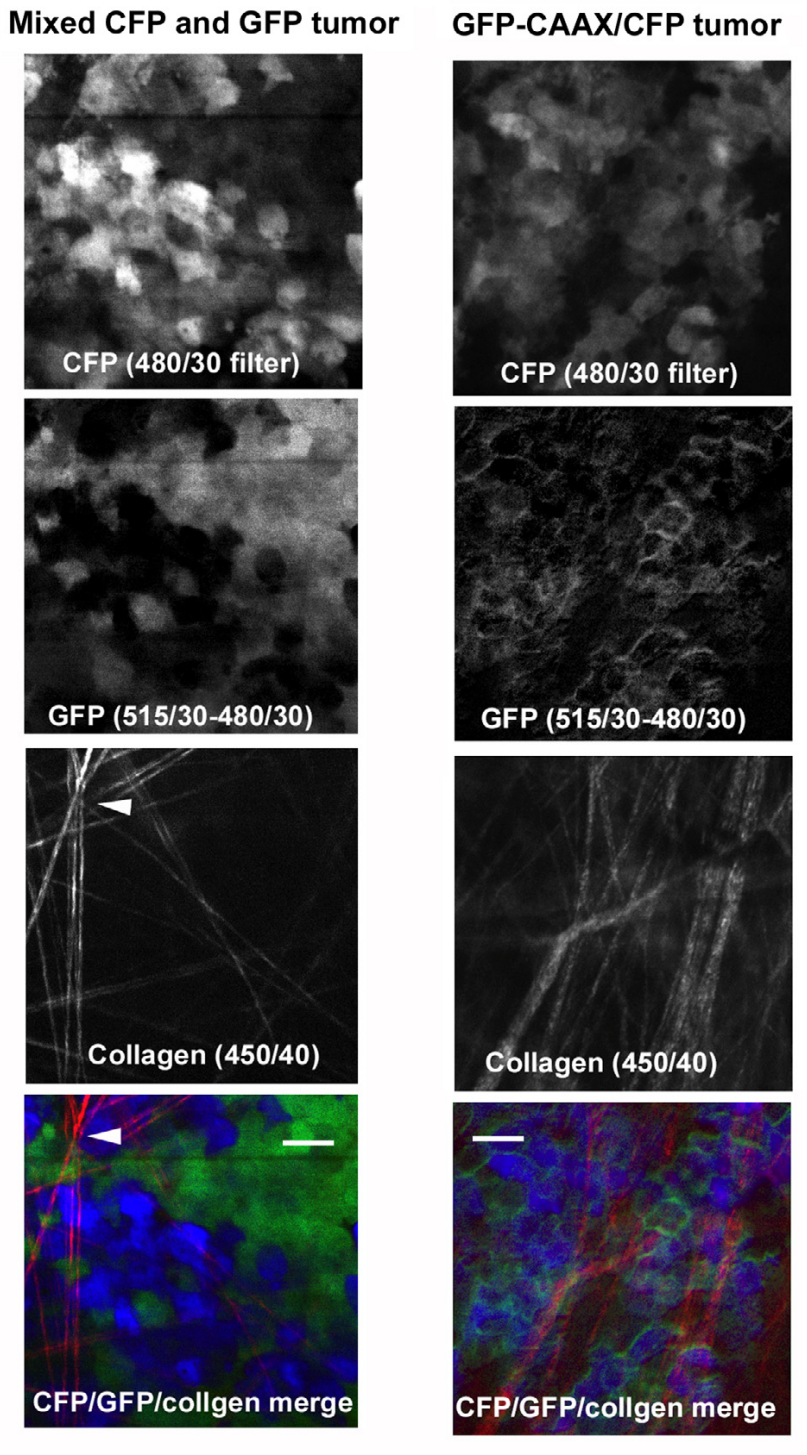
Simultaneous capture of GFP, CFP and collagen second harmonic fluorescence in a living tissue. The panels on the left-hand side show images of an experimentally generated mammary tumour creating by injecting a mixture of cells either expressing GFP or CFP. The panels on the right-hand side show images of an experimentally generated mammary tumour with cells expressing CFP to mark the entire cytoplasmic volume and GFP-CAAX (membrane targeted). 880 nm laser light was used to excite the all the samples and the fluorescence was captured with indicated filters using non-descanned detection. All images were taken with a 40× objective and a final magnification of 500×. Scale bar = 25 μm.

We next investigated if the method described above would work *in vivo*, we generated tumors composed of either a mixture of GFP and CFP expressing cells or cells co-expressing membrane targeted GFP and cytoplasmic CFP by co-injecting 5 × 10^5 ^cells of each of the mixed population or 10^6 ^cells that have GFP-tagged proteins into the mammary fat pads of SCID mice. An additional potential structure that can be imaged *in vivo *using multiphoton microscopy is the collagen fiber matrix using second harmonic fluorescence [20]. When using an excitation wavelength of 880 nm the second harmonic of collagen would be predicted to have a wavelength of around 440 nm which is only slightly shorter than CFP fluorescence. We found that second harmonic light generated by collagen fibers passed through the 450/80 filter but not the 480/30 (Figure 3 left hand panels – fibers marked with arrowheads). It was also able to pass through a 450/40 filter which CFP fluorescence was not able to pass through. Thus by using 450/40, 480/30 and 515/30 filters followed by subtraction of the 480/30 signal from the 515/30 we can simultaneously image collagen, CFP and GFP in a living tissue. The left hand panels in Figure 3 show this method used on a tumor consisting of a mixture of GFP and CFP expressing cells. The right hand panels show that this method can also be used to simultaneously image a membrane targeted GFP and CFP expressed in the same cell. A limitation in the past with imaging GFP tagged proteins *in vivo *has been determining the outline of the cell. By labeling the cytoplasm of the cell with CFP, we can now determine protein localization within a cell in a living tissue. Tumors generated using GFP-expressing cells alone or CFP-expressing cells alone show no differences in tumor growth and metastatic ability when compared to parental MTLn3 cells ([1] and data not shown)

**Figure 4:**
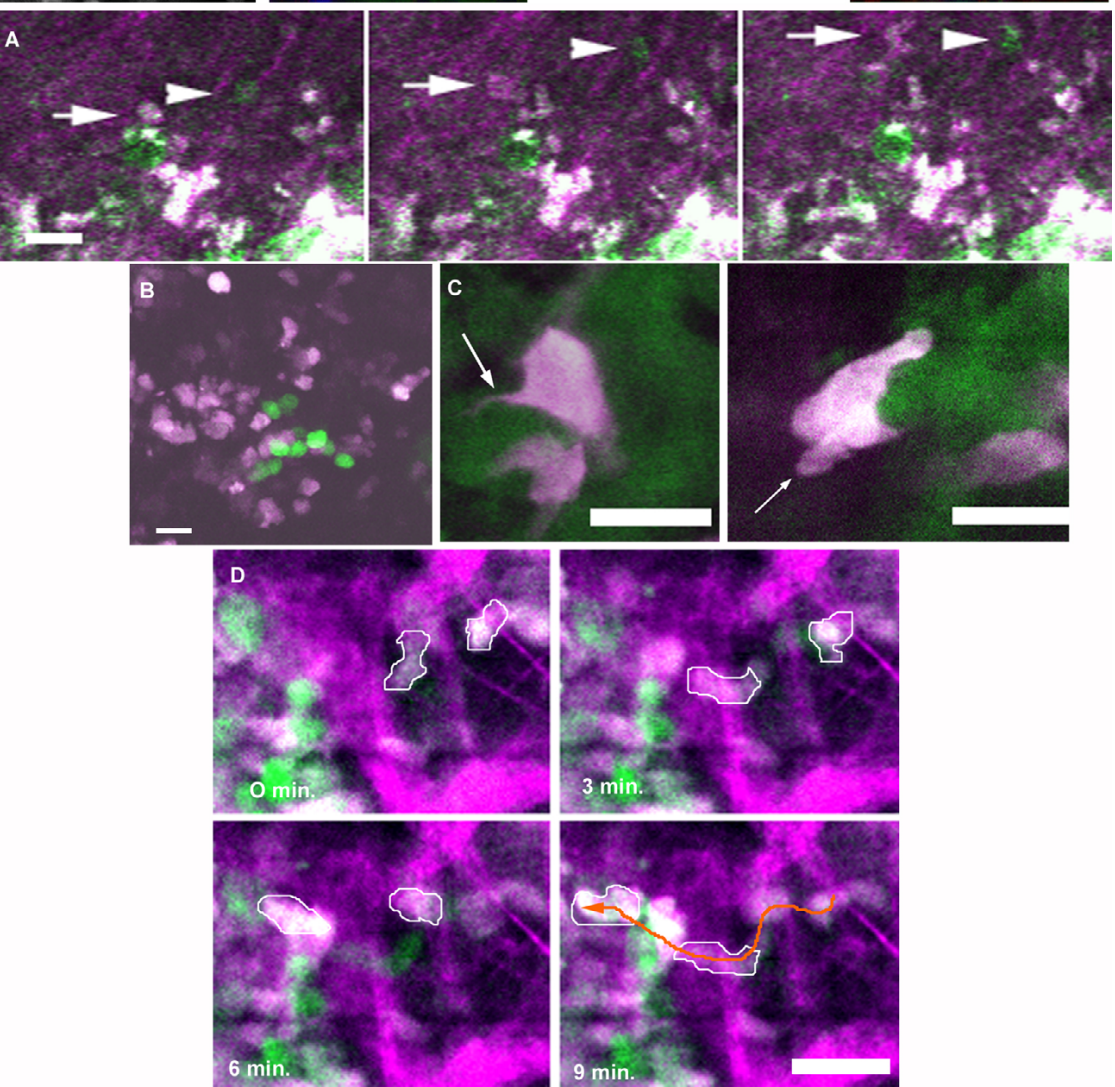
Differences in motility and invasion can be determined by imaging cells with different metastatic potential in the same tumor. A) The panels show a time series of images of an experimentally generated mammary tumour with cells either expressing GFP (low metastatic in green) or CFP (high metastatic in white) with collagen fibres in purple. Arrows point to moving CFP cells and arrowheads to moving GFP cells. Supplementary movie shows this image sequence including intermediate frames (Additional file 1). Images were taken with a 20× objective and a final magnification of 250×. B) CFP-labeled cells (white) and control GFP labelled cells (green) are seen in the lung after extravasation and metastatic growth. Images were taken with a 20× objective and a final magnification of 250×. C) Left hand panel CFP cell (white) has a filopod (arrow) protruding into a field of GFP control cells (green). Right hand panel CFP cell (white) has a lamellapod (arrow) seen in the middle of a field of GFP control cells (green). Supplementary movie shows a lamellapod retracting over time (Additional file 2). Images were taken with a 60× objective and a final magnification of 1000×. D) The panels show a time series of high magnification images of an experimentally generated mammary tumor with cells either expressing GFP (in green) or CFP (in white) with collagen fibers in purple. Moving high metastatic cells are outlined in white and an orange arrow shows the path taken by both cells. Supplementary movie shows this image sequence including intermediate frames (Additional file 3). Images were taken with a 40× objective and a final magnification of 500×. Scale bar for A-E = 25 um.

Past intravital imaging of cells in living tumors by our group was able to establish differences such as motility, protrusion, and, cell polarity and orientation [6,16] between non-metastatic and metastatic tumor cells. By comparing gene expression in tumors of differing metastatic ability we found that many genes regulating motility and invasion are dramatically changed in metastatic cells. Furthermore, using our *in vivo *invasion assay we demonstrated that the expression of these is further increased in the motile subset of metastatic tumor cells [6,21]. However, these studies were not able to determine if the altered metastatic potential of these tumors is due to non cell autonomous differences in the tumor environment or to cell autonomous changes in the behavior of individual tumor cells. To directly compare the behavior of cells with differing metastatic potential in the same tumor environment *in vivo*, we generated a tumor by injecting a mixture of cancer cells with low metastatic potential labeled with GFP and high metastatic potential labeled with CFP. These cells were derived from the same genetic background [21] and their metastatic potential was determined by counting lung metastases in animals with primary tumors derived from injection of these cells into the mammary gland as described previously [1,6,22]. Figure 4A shows a time-series in which a greater number of cells with high metastatic potential (shown in white) are seen moving compared to control (shown in green) along collagen fibers imaged by second harmonic generation (purple). A time lapse of this sequence can be found in supplemental data (Additional file 1). The number pixels for each cell type was calculated in each field and used to normalize for differences in cell number of either type of cell in any given field. These data are the first demonstration that cells with higher metastatic potential move more frequently, by about 4.5 fold (n = 12 animals), than low metastatic cells in the same tumor micro-environment.

Our multi-fluor technique can also be used to explore metastatic ability. While fluorescently labeled cell have been used to explore metastatic and extravasive ability of differing cell types [9,23,24], our technique allows for multiphoton imaging at single cell resolution within the primary tumor of two cell populations and determination of differences in metastatic potential within the same animal. By examining of the lungs of animals with tumors formed by the injection of a mixture of CFP and GFP cells, extravasated cells can be counted to determine differences in numbers of cells metastasizing and compare this score to cell behavior differences in the primary tumor. Figure 4B shows how both cell types can be visualized in the lungs.

To visualize dynamic structures involved in cell motility we used a 60× LUMPlan/IR 0.9NA dipping objective. The morphology of single cells labeled with either CFP or GFP was easily visible on a background of the opposing color. Cell structures that were not well defined in prior *in vivo *experiments using conventional microscopy [1] can now be easily discerned. Cells are seen with filopodia (Figure 4C, left) and lamellipodia (Figure 4C, right) which when visualized as time-lapse movies (Additional file 2) can be seen protruding and retracting. Morphological changes of moving cells can also be resolved. Analysis of these time-lapse movies revealed that motile cells moved with amoeboid characteristics with the edge moving at up to 8 μm/min and making rapid changes in direction (Figure 3E and Additional file 3). The speed of the highly metastatic cells were twice what was previously reported for tumor cells with average metastatic potential in vivo [22]. Furthermore, motile cells often followed the same path; such behavior has been inferred from the presence of chains of invading cells in fixed breast cancer samples [25] but this is the first direct observation of such behavior. These cells may follow the same path because it is physically favorable (i.e. lack of ECM blocking cell movement) or because of a chemotactic relay between the cells.

## Conclusion

Recent advances in multiphoton imaging technologies have provided researchers with the tools to study cell-cell interactions and the microenvironment of living tissues [4,5,26,27]. Our multi-fluor method described here will enable the direct comparison of the behavior of control and experimental manipulated cells in the same biological environment using multiphoton microscopy. This will be invaluable for determining the effects of genetic manipulations on cell behavior *in vivo*. In addition, the simultaneous imaging of GFP and CFP within the same cell will greatly aid the study of sub-cellular compartments and protein localization in living tissues.

## Methods

### Plasmids and Cell lines

ECFP-N1 (Clontech), GFP-CAAX (AP4-CAAX described [28]), GFP-zyxin [29]. Standard retroviral and transfection methods were used to generate the following derivatives of the MTLn3 rat mammary carcinoma cell line: low metastatic MTLn3-pLXSN GFP, high metastatic MTLn3-ECFP N1,0 MTLn3-GFPCAAX + ECFP N1, MTLn3-GFPZYXIN + ECFP N1. Cells were maintained in αMEM + 5% Fetal Calf Serum. Pools of GFP and CFP expressing cells were isolated by FACS methods. For imaging, cells were grown in MatTek dishes and fixed with 4% formaldehyde in PBS.

### Tumor analysis

To determine metastatic differences between the MTLn3 clones, a total of 10^6 ^cells were injected into the mammary fat-pad of groups of eight 5–6 week old female Balb/C SCID mice. After 4 1/2 weeks animals were euthanized and lung mets counted in H and E stained sections. To determine motility differences, cells were compared by using a Boyden Chamber chemotaxis assay. Both these experiments were performed as reported previously [6,22].

### Imaging

10^6 ^MTLn3 cells were injected into the mammary fat-pad of either female 5–6 week old female Balb/C SCID mice for the comparison of cell motility and metastatic potential or 5–6 week old Fischer 344 rats to image the cell membranes. After 3–4 1/2 weeks animals were anaesthetized with isoflurane and small incision was made in the skin to expose the tumor [1,6,26]. The anaesthetic was maintained while the tumor was imaged using a Biorad Radiance 2000 multiphoton microscope with an inverted Olympus IX70, within a heated chamber maintained at 30°c, connected to a Spectra Physics Tsunami Ti-Sapphire laser. All images were collected using non de-scanned detectors. The objectives used were as follows: 20× Plan Apo 0.7NA (air), 40× LUMPlan/IR 0.8NA (water) and 60× LUMPlan/IR 0.9NA (water). The filters used were as follows: 450/40, 450/80, 480/30, 515/30 (all from Chroma). Time lapsed images were taken over the course of 30 mins and analyzed using ImageJ. Frequency and speed of motility were calculated as described previously [22].

## Authors' contributions

ES and JW conceived of and designed the study, did all imaging, all mathematical and statistical analysis and drafted the manuscript. UP transfected and maintained all cells with GFP-tagged proteins. JS determined the metastatic potential of the cells used. FG aided in design of the study and drafting of the manuscript. JC participated in the study's design and coordination and helped to draft the manuscript.

## Supplementary Material

Additional File 1 Movie for figure 4A (avi. Format): Time-lapsed movie of high metastatic CFP labeled cells (white) and low metastatic GFP labeled cells (green) crawling on collagen fibers (purple) *in vivo*. Objective used 20×. Click here for file

Additional File 2 Movie for figure 4C (avi. Format): Time-lapsed movie of a CFP labeled cell with a ruffling lamella on a field of GFP labeled cells in a living tumor. Objective used 60×. Click here for file

Additional File 3 Movie for figure 4E (avi. Format): Time-lapsed movie of high metastatic CFP labeled cells (white) and low metastatic GFP labeled cells (green) crawling on collagen fibers (purple) *in vivo *showing amoeboid characteristics and making rapid changes in direction. Objective used 40×. Click here for file
